# Practices and opinions on nitrous oxide/oxygen sedation from dentists licensed to perform relative analgesia in Brazil

**DOI:** 10.1186/1472-6831-12-21

**Published:** 2012-07-18

**Authors:** Anelise Daher, Renata Pinheiro Lima Hanna, Luciane Rezende Costa, Cláudio Rodrigues Leles

**Affiliations:** 1 Health Science Program, Federal University of Goias, Goiania, Goias, Brazil; 2 Dental Service, Unified National Health System, Goiania, Goias, Brazil; 3 Department of Pediatric Dentistry, School of Dentistry, Federal University of Goias, 1a Avenida, s/n, Setor Universitário, 74605-220, Goiania, Goias, Brazil; 4 Department of Prevention and Oral Rehabilitation, School of Dentistry, Federal University of Goias, Goiania, Goias, Brazil

**Keywords:** Relative Analgesia, Nitrous Oxide, Dental Clinics, Cross Sectional Survey

## Abstract

**Background:**

Relative analgesia (RA), defined as the use of inhalation sedation with nitrous oxide and oxygen, is one of the most common pharmacological behavior management techniques used to provide sedation and analgesia for dental patients. This study aimed to assess RA licensed Brazilian dentists’ practices and opinions about nitrous oxide/oxygen sedation in the dental setting.

**Methods:**

A cross sectional national survey was conducted with 281 dentists who were certified to perform RA, using an electronically mailed self-administered questionnaire containing closed questions about their practices and opinions regarding RA. Practice and opinion were individually analyzed by descriptive statistics. Non-parametric tests assessed the relationships between RA practice and independent variables. To test the interplay between practices and opinions, a k-means clusters analysis was used to divide the group for statistical comparisons.

**Results:**

The response rate was 45.2%. Women made up 64.6% of the respondents, the mean age was 39.1 years (SD = 9.8), and the mean time since graduation in dentistry was 16 years (SD = 9.7). Seventy-seven percent of respondents reported the use of RA in clinical practice, most of them ‘sometimes’ (53.5%), and focusing more on adult patients. Patients with certain physical or mental deficiencies were indications associated with RA practice. ‘Equipment acquisition’ (p < 0.001) and ‘living in Southeast and South regions’ (p < 0.02) were also associated with RA practice. The scores for dentists’ opinions ranged from 15 to 41 points (mean 29.2, SD = 5.6), based on nine items scored from 1 to 5. Two clusters representing more favorable (n = 65) and less favorable (n = 55) opinions were established. Dentists who were women (p = 0.04), practiced RA in dental settings (p < 0.01) or practiced it frequently (p < 0.001), had more favorable opinions about RA.

**Conclusion:**

Most of the RA licensed Brazilian dentists interviewed currently use RA. Current practice of RA and frequency of use determined the degree of favorable opinion about this inhalation sedation among this group of respondents.

## Background

Relative analgesia (RA) is a term introduced in dentistry by the American Harold Langa, in 1968, to represent inhalation sedation with a continuous flow and variable concentrations of nitrous oxide and oxygen to produce sedation and analgesia [1]. Langa proposed three planes of analgesia within the first stage of anesthesia described by Arthur Guedel in 1937 [2]; those planes vary from moderate to total analgesia according to the concentration of nitrous oxide in the mixture, and the signs and symptoms shown by patients. The aim of RA is to help fearful and/or anxious patients feel more relaxed, thereby facilitating patient behavior management during medical and dental procedures.

In several countries, including the United States, Canada, Australia, New Zealand, and the United Kingdom, RA is a technique used in various medical specialties, including dentistry [3], and can be considered the most popular form of sedation among pediatric dentistry specialists [4]. Nevertheless, even in countries where dentists routinely use RA, lack of clinical experience and additional costs for purchasing the equipment may have an inhibitory effect on the practice of providing RA for children [5]. General dental practitioners in Northern Ireland, for example, had RA equipment in their practices in 29% of cases, and discussed RA as a treatment alternative for pediatric extractions with children and accompanying adults, but most preferred to refer patients for general anesthesia to have teeth extracted [5]. Although the costs for RA may be lower than general anesthesia or multiple drug sedation, RA is not recommended as an alternative for all cases referred for general anesthesia due to its particular indications and limitations. A systematic review did not find randomized clinical trials to support the cost-effectiveness of sedation versus general anesthesia for provision of pediatric dental treatment [6], but another study which analyzed the cost of time spent on the procedure found RA to be less expensive than general anesthesia for dental extractions in children [7].

In Brazil, the use of RA in dentistry was endorsed by the Brazilian College of Dentists (BCD) in 2004. Current legislation dictates that dentists are permitted to provide RA following a 96-hour training course and submitting proof of completion to the BCD. It is important to note that many Brazilian dentists have limited training and practice experience in outpatient sedation as part of dental school [8]. Criticism by anesthesiologists concerning the competency of dentists to provide outpatient sedation has been reported as a barrier that prevents RA practice among licensed dentists [9].

The purpose of this survey was to identify current practices and opinions of RA licensed Brazilian dentists about nitrous oxide/oxygen sedation for dental patients.

## Methods

### Study design and sample

This was a cross sectional survey of RA licensed dentists in Brazil that was approved by the Institutional Research Board of the Federal University of Goias, Brazil. According to the BCD web site, there were 652 licensed dentists able to provide RA in 2007. Eligible dentists were those who formally presented the RA training course conclusion certificate to the BCD (complete names available at the BCD web site). Dentists’ names were used to search for their electronic addresses and/or telephone numbers via internet tools (Google, social networking services, and a resume database). From 652 eligible dentists, 305 were excluded because their contact information was not available online. From the remaining 347 licensed dentists, 62 did not have a valid electronic address (automatic message “mail returned to sender”) and 4 refused to participate. The final sample comprised 281 dentists who agreed to participate in the study answering back the first email sent (43.1% of eligible dentists).

### Questionnaire development

For construction of the self-administered questionnaire, a series of individual in-depth interviews were previously conducted with six dentists trained in RA. The interviewer used an interview guide containing a list of sequenced key questions in conversational sentences, including topics about: the RA training course, licensing process, equipment acquisition, frequency of equipment use, factors influencing RA practice, and level of satisfaction with RA. Interviews were audiotaped and then transcribed verbatim into an electronic text editor for subsequent analysis.

Data analysis consisted of examining and categorizing all relevant information that represented a common viewpoint or perspective connected to the key questions or purpose of the study. A content analysis method was used to identify themes that emerged from the data, and was used as an item generation procedure for the construction of a questionnaire for quantitative analysis.

A preliminary version of the questionnaire was reviewed by three research consultants and, after minor changes, was tested on a group of 16 dentists trained to perform RA. The final instrument consisted of two parts, including demographic characteristics and information on RA practice, and opinions of respondents about RA. None of the 22 respondents from the questionnaire development steps participated in the final data collection phase.

A list of indications and contraindications adapted from the American Academy of Pediatric Dentistry guidelines [10] was provided, and respondents were asked to mark as many indications and contraindications for RA as they considered suitable in their professional practice, and add any other new items they would suggest as an indication or contraindication for RA.

Part 2 of the questionnaire explored the opinions of respondents about RA. Nine items were answered according to a 5-point Likert scale, with scores ranging from 1 to 5, in which a score of 1 indicated strong disagreement and a score of 5 indicated strong agreement with the affirmatives. We obtained the total score of the questionnaire by adding the scores of these nine statements. The highest possible score was 45 points (multiplication of the highest score ‘5’ by the number of statements ‘9’) and would represent the most positive opinions about RA; the lowest score possible was 9 points (multiplication of lowest score ‘1’ by the number of statements ‘9’) and meant unfavorable opinions about RA. Six out of the 9 statements were phrased in the reverse, which means that strong agreement indicated more negative opinions about RA; in order to make those items comparable to the other items, we had to reverse score them for the statistical analysis.

### Data collection and analysis

A cover letter explaining this study’s purpose and an informed consent form, including information regarding the confidentiality of the responses, was sent electronically. If a dentist sent an email back agreeing to participate, the self-administered questionnaire used for data collection was sent. Documents were sent as an attachment file or in the body of the email, depending on the respondent’s preference. Questionnaires were returned by email.

Responses were entered in a database, and statistical analysis was performed using Statistical Package for Social Science 19.0 (SPSS Inc., Chicago, IL, USA). Descriptive analyses were performed for demographic data. We initially considered RA practice as a dependent variable, and tested associations with independent variables using non-parametric Mann–Whitney U and chi-square tests.

To study dentists’ opinion about RA, frequency distributions of the ratings’ scores (Likert scale) and means of scores were included; Cronbach’s alpha coefficient was checked to investigate the internal consistency of Part 2 of the questionnaire. Using a proposed model [6], a k-means cluster analysis (non-hierarchical model) was performed to divide answers about dentists’ opinions into 2 clusters denoted as “less favorable” and “more favorable” opinions. As the continuous cluster variables followed a normal distribution (p = 0.06, Kolmogorov-Smirnov test), the Student’s *t*-test was used to compare means of scores between the groups, and with the continuous independent variables (age and length of time since graduation). The Chi-square test was used to compare the two clusters in regard to the other independent variables (gender, region of practice, equipment acquisition, RA practice, and frequency of RA practice). Statistical significance was set at p < 0.05.

## Results

A total of 136 participants sent back the email. Among these, 9 respondents had inadequately filled out the questionnaires and were excluded from the final analysis (response rate = 45.2% out of the 281 dentists contacted who agreed to receive the questionnaire).

### Respondents’ characteristics and RA practice

Most of the respondents were women (64.6%, 82 out of 127) and worked in South or Southeast regions of the country (80.4%, 102 out of 127). The participants’ mean age was 39.1 years (range 24–72 years, SD = 9.8), and the mean length of time since graduation was 16.0 years (range 3–50 years, SD = 9.7).

Licensed dentists were diverse in their practices, and 77.2% (98 out of 127) currently used RA (Table 1). Moreover, most of them had acquired RA equipment (88 out of 126, 69.8%); others had not acquired it (18 out of 126, 14.3%) or intended to acquire sometime in the future (n = 20 out of 126, 15.9%). Regarding other approaches to sedation cited by 70 respondents, two modalities were cited most often: (1) oral sedation with benzodiazepines or chloral hydrate (40 out of 127, 31.5%); (2) intravenous drugs provided by an anesthesiologist in a dental or hospital setting (25 out of 127, 19.7%).

**Table 1:** Characteristics of the practices of Brazilian dentists licensed in relative analgesia

**Characteristics**	**N**	**%**
**Practice**		
More than two specialization degrees	28	22.2
Pediatric Dentistry	28	22.2
Oral and Maxillofacial Surgery	23	18.3
Implant Dentistry and Periodontology	17	13.5
General Dentistry	16	12.7
Other specialization	14	10.4
Did not answer	1	0.8
**Population served by relative analgesia**		
Adults and children	52	40.9
Adults	34	26.8
Children	7	5.5
Did not answer	34	26.8
**Practice of relative analgesia**		
During training program and after	102	80.3
In training program only	23	18.1
None	2	1.6
**Practice of relative analgesia in respondents’ own dental practice**		
Yes	90	70.9
No	36	28.4
Did not answer	1	0.8
**Frequency of practice of relative analgesia (**includes respondents’ use in dental practices other than their primary practice location**)**		
Never	27	21.3
Sometimes	68	53.5
Often	18	14.2
Always	12	9.4
Don’t know	1	0.8
Did not answer	1	0.8

**Figure 1:**
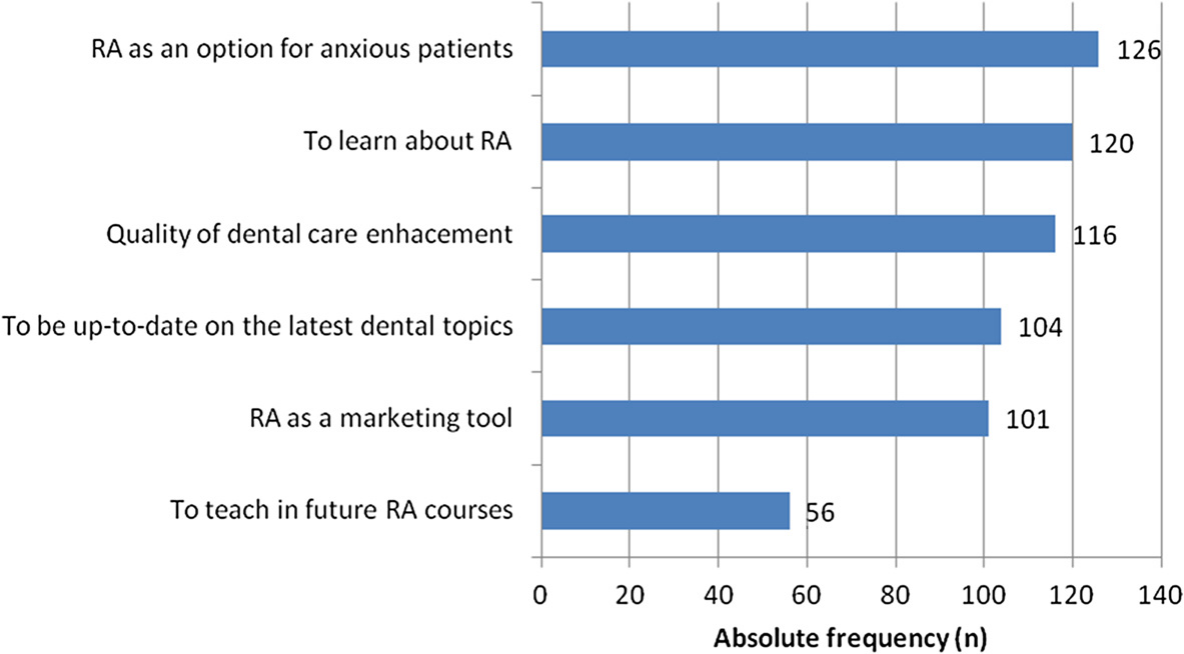
Reasons chosen by respondents to attend a relative analgesia (RA) training course.

According to the respondents, the main reason for attending an RA training course was to offer an option for dentally anxious patients (Figure 1).

Among the circumstances written in the questionnaire as possible indications or contraindications for RA, the most of participants had similar opinions about RA recommendation (Table 2). Physically (p = 0.01) or mentally (p = 0.02) compromised patients were the only indications clearly associated with those dentists who practiced RA in the dental office.

**Table 2:** Indications and contraindications of RA between dentists who do or do not practice RA*

	**RA practice n (%)**		**Total n (%)**
	**Yes**	**No**	126 **(100.0)
	**n = 90**	**n = 36**	
**Indications of RA**			
Fearful patients	88 (97.8)	34 (94.4)	122 (96.8)
Anxious patients	84 (93.3)	33 (91.7)	117 (92.8)
Disruptive patients	47 (52.2)	19 (52.8)	66 (52.4)
Physically compromised patients***	53 (58.9)	12 (33.3)	65 (51.6)
Mentally compromised patients***	45 (50.0)	10 (27.8)	55 (43.6)
Medically compromised patients	32 (35.6)	13 (36.1)	45 (35.7)
Gag reflex interfering with dental care	33 (36.7)	11 (30.6)	44 (34.9)
Ineffective local anesthesia	34 (37.8)	8 (22.2)	42 (33.3)
Long appointments for dental care	20 (22.2)	9 (25.0)	29 (23.0)
**Reasons for RA contraindications**			
Chronic obstructive pulmonary disease	83 (92.2)	32 (88.9)	115 (91.2)
Several emotional disturbances	73 (81.1)	27 (75.0)	100 (79.3)
Drug-related addiction	64 (71.1)	27 (75.0)	91 (72.2)
First semester of pregnancy	59 (65.6)	23 (63.9)	82 (65.1)
Treatment with bleomycin sulfate	41 (45.6)	17 (47.2)	58 (46.0)

* More than one alternative was allowed.

** One questionnaire considered missing was excluded from the analysis.

*** Significant difference between groups at the P < 0.05 level.

The region of a dentists’ practice was also associated with RA practice (p = 0.02, one case missing): 76.2% (n = 77 out of 101) of dentists working in the South/Southeast regions used RA in their current practice, against 52.0% (n = 13 out of 25) working in less wealthy geographic regions (Midwest and Northeast). The acquisition of RA equipment, dichotomized in ‘yes’ or ‘no’ answers, was also associated with RA practice (p < 0.001, two cases missing) with 93.1% (81 out of 90) of those who acquired the equipment reporting RA practice. Other variables were not associated with RA practice: gender (p = 0.19), those who reported a pediatric practice (p = 0.46), time since graduation (p = 0.92), those acting in private practice (p = 0.08), or the age of participants (p = 0.93).

### Respondents’ opinions about RA

Table 3 shows the frequencies of respondents’ opinions about RA practice. The mean total score for the nine statements in Part 2 of the questionnaire was 29.2 (SD = 5.6, range 15–41), indicating the opinion of RA by respondents was slightly positive. Respondents’ opinions on RA varied according to the statements proposed, but they generally followed a pattern of agreement or disagreement, except for the item about anesthesiologists’ opinion of dentists practicing RA; that item showed a balance between agreement and disagreement (Table 3).

**Table 3:** Frequencies of respondents’ opinions about RA practice, measured on a 5-point Likert scale (scores 1 to 5)

	**Absolute frequencies**					**Scores’ mean (SD)**	**Did not answer (n)**
	**1** **Strongly disagree**	**2** **Disagree**	**3** **Neutral**	**4** **Agree**	**5** **Strongly agree**		
Patients/parent are satisfied with relative analgesia.	3	1	10	41	70	4.3 (1.0)	2
I am satisfied with the outcomes of relative analgesia.	3	5	21	36	61	4.2 (1.1)	1
The cost of the relative analgesia equipment is a problem to purchase it. *	8	8	11	32	64	4.0 (1.4)	4
Relative analgesia is effective for my patients.	5	9	16	48	49	4.0 (1.1)	0
Brazilian dentists’ acceptance of relative analgesia complicates its use because of cultural aspects.*	17	9	19	55	27	3.5 (1.3)	0
Patients/parents’ acceptance of relative analgesia complicates its use because of cultural aspects.*	18	19	16	47	26	3.3 (1.4)	1
Cost of relative analgesia could hinder acceptance by patients/parent.*	19	23	20	44	21	3.2 (1.3)	0
Brazilian anesthesiologists’ opposite opinions on relative analgesia performed by dentists inhibits it use.*	32	19	14	37	25	3.0 (1.5)	0
Environmental risk of nitrous oxide could be a limiting factor for the use of relative analgesia.*	60	27	21	15	4	2.0 (1.2)	0

* Scores were reversed for calculation of Cronbach’s alpha, and the sum of the scores.

Cronbach’s Coefficient Alpha = 0.64.

Cluster analysis divided the differences of opinion into two groups: (1) ‘less favorable’ opinions (n = 55), with total scores ranging from 15 to 28 (mean = 24.4, SD = 3.0); and (2) ‘more favorable’ (n = 65), with total scores ranging from 29 to 41 (mean = 33.3, SD = 3.6). Seven questionnaires were excluded from this analysis because they had incomplete items in Part 2. Dentists who practiced RA (p < 0.01) and were women (p = 0.04) had a more favorable opinion about this inhalation sedation. The frequency of RA practice was also associated with the dentists’ opinions, showing that those who practiced frequently had a more positive opinion (p < 0.001) (Table 4).

**Table 4:** Association between dentists’ opinion and independent variables

**Independent variables**	**Dentists’ opinion**^**(a)**^		p*
	**Less favorable**	**More favorable**	
Gender, n (%)			0.04
*Female*	30 (39.0%)	47 (61.0%)	
*Male*	25 (58.1%)	18 (41.9%)	
Region of practice, n (%)			0.36
*Southeast or South*	42 (43.3%)	55 (56.7%)	
*Midwest, North or Northeast*	13 (56.5%)	10 (43.5%)	
Equipment acquisition**, n (%)			0.65
*Yes*	37 (44.0%)	47 (56.0%)	
*No*	17 (48.6%)	18 (51.4%)	
RA practice**, n (%)			<0.01
*Yes*	31 (36.0%)	55 (64.0%)	
*No*	23 (69.7%)	10 (30.3%)	
Frequency of RA practice**, n (%)			<0.001
*Low*	50 (55.6%)	40 (44.4%)	
*High*	5 (17.9%)	23 (82.1%)	
Age (yr), mean (SD)	38.8 (8.0)	39.2 (10.7)	0.07
Length of time since graduation (yr), mean (SD)	15.8 (8.0)	15.9 (10.6)	0.06

^(a)^ Dentists’ opinions were divided into two groups (cluster analysis); 7 questionnaires were excluded from this analysis because they had incomplete items in Part 2.

*Chi-Square test and Student’s *t*-test.

** Variables with missing data because several participants did not answer the item.

## Discussion

This survey sought to profile RA practice within a group of RA licensed Brazilian dentists who were mostly specialists, primarily focused on treatment of adults, and who for the most part practiced RA “sometimes”. According to our results, the dentists’ opinions were strongly related to details of their practices.

The practice of RA determined the favorability of opinions about this kind of sedation. Agreeing that attitudes and beliefs were predictors of the behavior ‘intention to provide RA’ [5], this study showed that a higher frequency of RA practice positively influenced dentists’ favorable opinions.

Most of the respondents reported they attended a training course in order to offer patients a choice for dental anxiety control. The most cited indications for RA were fearful and/or anxious patients, but the only indications that were significantly associated with RA practice after bivariate analysis were recommending RA for patients with certain mental or physical deficiencies. As these kinds of deficiencies are easily identifiable, we suspect this group of dentists do not actually use any systematic strategy to diagnose dentally anxious patients, and so are unable to recognize a patient with low to moderate anxiety. Possibly only patients who clearly demonstrated their anxiety would be offered RA. This suspicion is also supported by other studies [11] which found that dental anxiety level is a good predictor of referral for sedation; that is, highly anxious patients were more likely to be referred for sedation.

We did not find any differences in RA practice regarding its use in adults or children. On the contrary, surveys in other countries have shown that pediatric dentists are the specialists who use RA the most [12-14], and this modality is very popular among them [15,16]. Thus, there is a trend for expansion of RA into other specialties that treat adults [17-19]. The American Dental Association advocates that an RA course should be a minimum of 14 hours, completed as a part of the predoctoral dental education program or in a postdoctoral continuing education competency course [20]. In addition, a group of Canadian dentists believe RA should be included in the treatments that a licensed general practitioner can provide [21].

In this study, the practice of RA was significantly associated with the region of practice and the acquisition of the equipment. First, dentists practicing RA are concentrated in the South and Southeast Brazil, raising the prospect of an existing tendency for polarization in RA practice, perhaps because most of the qualified dentists live in these regions, and they are located where there are more RA training courses. Second, those who acquired the equipment were more easily able to practice RA, according to another study conducted in Northern Ireland [5], where those dentists that did not have RA equipment available in their practice were less likely to offer RA for pediatric extractions.

Respondents in this study generally agreed that RA has positive aspects, including its effectiveness, and satisfaction from both patients and professionals. Literature on the use of RA during dental treatment reports its usefulness in both children [22] and adults [18,19]. The majority of a sample of 100 Italian preschool children appreciated RA and would like to have it offered again in their next sessions [23]. Participants in this study reported one of the disadvantages of RA is that its acceptance by professionals and patients depends on cultural aspects and costs. In fact, nitrous oxide is one of the least accepted techniques by Kuwaiti parents, because the use of pharmacological techniques can be perceived as risky in that culture [24]. Regarding the costs of RA, it is less expensive than general anesthesia [7] and probably other multidrug sedation, but carries an initial charge for the dentist to purchase the equipment.

Interestingly, respondents were unaware of the occupational risks of nitrous oxide. According to the literature, this is one of the most commented on points related to RA that limits its use [25]; chronic exposure to high levels of ambient nitrous oxide presents health hazards for dental personnel and patients which can have reproductive, hematologic, immunologic, neurologic, hepatic, and renal impacts [26]. Occupational exposure to nitrous oxide can be controlled by effective vacuum gas-scavenging systems included in RA equipments, as well as by good work practices such as appropriate mask size selection and mask adjustment, minimal talking and mouth breathing by the patient [27].

In Brazil, one study showed that 93.7% of anesthesiologists surveyed disagreed that licensed dentists are adequately prepared to provide RA after the 96-hr training course required by the BCD [9]. However, this understanding of anesthesiologists’ opposition was not a clear barrier to RA practice among Brazilian respondents, since only about half the respondents agreed that anesthesiologists’ contrary opinion on RA should limit its use by dentists. Moreover, a recent trial (ENIGMA trial) performed with anesthesiologists about the usage of nitrous oxide for general anesthesia were reported in three studies showing both positive [28] and negative [29,30] recommendations in different situations.

In general, the sum of the scores reached by the respondents in our study represented an average level; that is, dentists in this study did not show the most positive opinions about RA. In another study [8], the level of knowledge about sedation was directly proportional to being in favor of its use and to the notion of associated risks. Perhaps more extensive practice with the RA technique during the training course could help dentists feel more secure about this sedation procedure, and have more positive opinions about it. Otherwise, it was reported that dentists’ perceptions of nitrous oxide inhalation sedation were generally less enthusiastic than those of patients and caregivers [31].

We recognize that this study as a survey had a major limitation in the coverage and non-response rate. Although we sent the questionnaire to all RA licensed dentists with available electronic mail, our response rate did not reach 50% of the study population. This response rate could be considered low for a survey targeting RA licensed dentists working in the whole country, but this is expected in electronically mailed questionnaires [32]. There have been other studies with a similar purpose which had low response rates of 47% [33] and 16% [16]. We understand that, as in another study with Brazilian health professionals [9], factors influencing response rates might include an unwillingness to participate or lack of interest in the subject. Also, we did not include dentists who attended a RA course but did not ask for their BCD license. In fact, the interpretation of the results should be viewed with caution because they primarily represent opinion rather than generalizable conclusions, as stated in another opinion study of professionals [16].

## Conclusions

A majority of the respondents practice relative analgesia ‘sometimes’ and have a fairly positive opinion about it. Although there may be questions about the theoretical criteria which indicate the technique, this group of RA licensed dentists had more favorable opinions if they performed RA as part of their routine practice.

There is a need to provide more comfortable treatment for dental patients. It is the author’s recommendation that the concerns discussed herein should be addressed by RA-training course directors, especially in locations where the use of RA for dental treatment is not well-established or commonly practiced.

## Competing interests

The authors declare that they have no competing interests.

## Authors’ contributions

AD performed statistical analysis, participated in writing the manuscript, and in the final revision. RPLH participated in the research design, carried out participant interviews, and all data collection. LRC supervised the work from the beginning, participated in the research design, statistical analysis, in writing the manuscript, and in the final revision. CRL participated in the statistical analysis of the data and in the final revision. All authors read and approved the final manuscript.

## Pre-publication history

The pre-publication history for this paper can be accessed here:

http://www.biomedcentral.com/1472-6831/12/21/prepub
